# No effect of resveratrol supplementation after 6 months on insulin sensitivity in overweight adults: a randomized trial

**DOI:** 10.1093/ajcn/nqaa125

**Published:** 2020-06-03

**Authors:** Marlies de Ligt, Maaike Bergman, Rodrigo Mancilla Fuentes, Hans Essers, Esther Moonen-Kornips, Bas Havekes, Vera B Schrauwen-Hinderling, Patrick Schrauwen

**Affiliations:** Department of Nutrition and Movement Sciences, NUTRIM School for Nutrition and Translational Research in Metabolism, Maastricht University Medical Center, Maastricht, Netherlands; Department of Nutrition and Movement Sciences, NUTRIM School for Nutrition and Translational Research in Metabolism, Maastricht University Medical Center, Maastricht, Netherlands; Department of Nutrition and Movement Sciences, NUTRIM School for Nutrition and Translational Research in Metabolism, Maastricht University Medical Center, Maastricht, Netherlands; Department of Nutrition and Movement Sciences, NUTRIM School for Nutrition and Translational Research in Metabolism, Maastricht University Medical Center, Maastricht, Netherlands; Department of Nutrition and Movement Sciences, NUTRIM School for Nutrition and Translational Research in Metabolism, Maastricht University Medical Center, Maastricht, Netherlands; Department of Internal Medicine, Division of Endocrinology, Maastricht University Medical Center, Maastricht, Netherlands; Department of Radiology and Nuclear Medicine, NUTRIM School for Nutrition and Translational Research in Metabolism, Maastricht University Medical Center, Maastricht, Netherlands; Department of Nutrition and Movement Sciences, NUTRIM School for Nutrition and Translational Research in Metabolism, Maastricht University Medical Center, Maastricht, Netherlands

**Keywords:** resveratrol, insulin resistance, intrahepatic lipid content, glycemic control, obesity

## Abstract

**Background:**

Effects of resveratrol on metabolic health have been studied in several short-term human clinical trials, with conflicting results. Next to dose, the duration of the clinical trials may explain the lack of effect in some studies, but long-term studies are still limited.

**Objectives:**

The objective of this study was to investigate the effects of 6-mo resveratrol supplementation on metabolic health outcome parameters.

**Methods:**

Forty-one overweight men and women (BMI: 27–35 kg/m^2^; aged 40–70 y) completed the study. In this parallel-group, double-blind clinical trial, participants were randomized to receive either 150 mg/d of resveratrol (*n* = 20) or placebo (*n* = 21) for 6 mo. The primary outcome of the study was insulin sensitivity, using the Matsuda index. Secondary outcome measures were intrahepatic lipid (IHL) content, body composition, resting energy metabolism, blood pressure, plasma markers, physical performance, quality of life, and quality of sleep. Postintervention differences between the resveratrol and placebo arms were evaluated by ANCOVA adjusting for corresponding preintervention variables.

**Results:**

Preintervention, no differences were observed between the 2 treatment arms. Insulin sensitivity was not affected after 6 mo of resveratrol treatment (adjusted mean Matsuda index: 5.18 ± 0.35 in the resveratrol arm compared with 5.50 ± 0.34 in the placebo arm), although there was a significant difference in postintervention glycated hemoglobin (HbA1c) between the arms (*P* = 0.007). The adjusted means showed that postintervention HbA1c was lower on resveratrol (35.8 ± 0.43 mmol/mol) compared with placebo (37.6 ± 0.44 mmol/mol). No postintervention differences were found in IHL, body composition, blood pressure, energy metabolism, physical performance, or quality of life and sleep between treatment arms.

**Conclusions:**

After 6 mo of resveratrol supplementation, insulin sensitivity was unaffected in the resveratrol arm compared with the placebo arm. Nonetheless, HbA1c was lower in overweight men and women in the resveratrol arm. This trial was registered at Clinicaltrials.gov as NCT02565979.

See corresponding editorial on page 905.

## Introduction

In Western society, the number of people suffering from age-related chronic diseases such as obesity and type 2 diabetes mellitus (T2D) has been increasing progressively (1). One of the most potent nonpharmacological interventions known to alleviate these deleterious conditions is caloric restriction. Although dietary restriction in humans has metabolic effects such as improving insulin sensitivity (2) and reducing cardiovascular risk markers (3), eating less in favor of creating a desirable metabolic profile is not likely to gain widespread compliance (4). As such, dietary restriction mimetics, that evoke some of the beneficial effects of calorie restriction without an actual reduction in energy intake, are being explored.

Resveratrol, a natural polyphenol present in various food items, was identified as a promising activator of sirtuin 1 (SIRT1) (5). SIRT1 is a member of a family of NAD^+^-dependent histone deacetylases that play a central role in health span regulation by mediating effects in metabolic stress situations such as high-fat diet-induced obesity as shown in mice (6). Therefore, compounds that activate SIRT1 directly or indirectly might offer protection against the onset of metabolic dysregulation and promote healthy aging.

The potential of resveratrol has been extensively studied in animals. These studies confirmed that resveratrol is indeed beneficial for metabolic health by improving insulin sensitivity, decreasing liver fat accumulation, and improving skeletal muscle mitochondrial function (7). However, results from human clinical trials are inconsistent. Some studies show clear enhancements in parameters related to metabolic health (8–13), whereas others find no or minimal effects (14–21). Conversely, studies in overweight and obese individuals and patients with T2D—employing extensive measurements of muscle metabolism—consistently show improvements in muscle mitochondrial oxidative capacity upon several weeks of low-dose resveratrol supplementation (8, 14, 22, 23), despite discrepancies in improvements in glucose homeostasis. Whether resveratrol supplementation can also affect whole-muscle functioning has only been investigated in 2 human clinical trials with mixed results (24, 25) and therefore warrants further investigation. Why some studies do and others do not find effects of resveratrol on metabolic parameters is unknown. It may involve factors such as dosage and duration of the resveratrol treatment. Treatment duration applied in most human clinical trials may be too short to translate improvements in mitochondrial function into effects on whole-body metabolic health. Long-term supplementation studies could therefore provide an important bridge between findings from animal studies and those from human studies. Therefore, the aim of the current study was to investigate the effects of long-term resveratrol supplementation on metabolic health outcome parameters in overweight men and women. The main objective of this study was to determine if insulin sensitivity, as defined by the Matsuda index, is higher after 6 mo of 150 mg resveratrol per day compared with 6 mo of placebo. A dose of 150 mg/d was chosen because we have previously and consistently shown that this dose can improve muscle mitochondrial function (8, 14, 23).

## Methods

### Participants

Overweight and obese men and postmenopausal women were recruited between April 2016 and August 2018. Inclusion criteria required a BMI (in kg/m^2^) between 27 and 35 and ages between 40 and 70 y for men and 50 and 70 y for women. Upon screening, it was verbally verified if women were postmenopausal, as defined by not having a menstrual period for ≥12 mo. Additional inclusion criteria were a stable body weight (no weight gain or loss >5 kg in the past 3 mo) and willingness to limit consumption of resveratrol-containing food products and refrain from resveratrol-containing nutritional supplements. Main exclusion criteria were uncontrolled hypertension, glycated hemoglobin (HbA1c) >48 mmol/mol, diagnosed with T2D, alcohol consumption >20 g/d, use of medication known to interfere with glucose homeostasis/metabolism, and participation in another biomedical study within 1 mo prior to start of the intervention. A flowchart of the enrollment process is shown in **Supplemental Figure 1**.

### Study overview

In this parallel-group, double-blind, randomized, placebo-clinical trial, 6 mo of resveratrol supplementation was compared with 6 mo of placebo. The protocol was reviewed and approved by the medical ethics committee of Maastricht University and Medical Centre (NL53016.068.15) and registered at Clinicaltrials.gov (NCT02565979). Eligible subjects started with a 2-wk run-in period during which they were instructed to stop consuming resveratrol-rich food items such as grapes, red wine, and peanuts. Participants adhered to these restrictions during the whole intervention period. After the run-in period, participants were randomly assigned (1:1 ratio) to 6 mo of 150 mg/d of *trans*-resveratrol (resVida, 99.9%; provided by DSM Nutritional Products) or 6 mo of placebo. The randomization scheme was generated by an independent researcher within the research group (online randomizer: http://randomization.com). For randomization, the participants were stratified for gender and age into 3 groups: men 40–55 y, men 56–70 y, and women 50–70 y. Participants and study personnel were blinded to experimental conditions using coding: A or B. Participants were allocated to either group A or group B, representing resveratrol or placebo treatment, respectively. Resveratrol and placebo supplements were identical in appearance, and containers were labeled with either the letter A or the letter B. Participants were instructed to consume 2 supplements of 75 mg resveratrol/placebo per day—1 during lunch and 1 during dinner.

At the beginning of the treatment period, on 2 separate days, baseline measurements were performed at Maastricht University (preintervention measurements). Measurements were repeated again at the end of the 6-mo treatment period on 2 separate days (postintervention measurements). The general experimental design is presented schematically in **Supplemental Figure 2**. During the intervention period, participants came to the university once a month in the morning in overnight fasted state. During these visits, body weight and blood pressure (Omron Healthcare) were measured, and a blood sample was obtained for the analysis of plasma resveratrol. The latter was analyzed by MS by DSM Nutritional Products as previously described (8). Finally, during these monthly visits, the participants received a new container of supplements for the upcoming month.

### Diet and physical activity standardization

To minimize meal effects on the metabolic health outcome parameters, all participants consumed the same standardized meal the evenings before the pre- and postintervention measurements (559 kcal, 73 g carbohydrates, 16 g fat, and 26 g protein). The participants consumed the meals at approximately the same time and remained fasted until the oral-glucose-tolerance test (OGTT) the next morning. In addition, they were instructed to avoid intense physical activity 2 d prior to these visits. Changes in physical activity level were monitored throughout the study: at baseline, after 3 mo, and after 6 mo physical activity level was assessed by Baecke's habitual physical activity questionnaire (26).

### Blood sampling

Blood samples were collected between 07:30 and 09:00 after an overnight fast. Pre- and postintervention, general safety parameters were determined: urea, creatinine, bilirubin, aspartate aminotransferase (AST), and alanine aminotransferase (ALT). In addition, markers for dyslipidemia (total cholesterol, HDL cholesterol, LDL cholesterol, triglyceride, and free fatty acid) and glucose homeostasis (glucose, insulin, and HbA1c) were measured.

### Insulin sensitivity

Pre- and postintervention, the primary outcome insulin sensitivity variable was assessed by calculating the Matsuda index from a 2-h OGTT. Given the high intraindividual variability in OGTTs, the OGTT was performed twice preintervention and also twice postintervention, separated by 2–6 d, and outcomes were averaged. A standard 2-h 75-g (Novolab), 5-point OGTT was performed, and blood samples were collected at time points 0, 30, 60, 90, and 120 min for measurement of plasma glucose. Samples were collected at time points 0, 60, and 120 min for plasma insulin. During OGTT, participants remained seated without moving, eating, or drinking. The Matsuda index was calculated as an estimate of insulin sensitivity, using glucose and insulin values of time points 0, 60, and 120 min (27). The average of the duplicate Matsuda index values was used for statistical analysis. In addition, glucose and insulin AUC were calculated by the trapezoidal rule. Incremental AUC (iAUC) was calculated by subtracting fasting glucose and insulin values.

### Intrahepatic lipid content

Pre- and postintervention, intrahepatic lipid (IHL) content was quantified by proton magnetic resonance spectroscopy (^1^H-MRS). The MRS scans were performed on a 3-T whole-body scanner (Achieva 3T-x; Philips Healthcare) in the morning in the overnight fasted state as described previously (28). In short, a voxel 20 × 20 × 20 mm was placed in the lower right hepatic lobe and a PRESS sequence was used with a repetition time of 4 s, an echo time of 32.5 ms, and 64 signal averages with frequency-selective number of prepulses for water suppression. A separate acquisition without water suppression and 32 signal averages, but otherwise identical settings, was performed to determine the signal intensity of the water resonance. Values are given as T2 corrected (29) ratios of the CH_2_ peak relative to unsuppressed water resonance, expressed as percentage. Spectra were fitted with a home-written script (30) in MATLAB R2014b (MathWorks).

### Body composition

Total mass, fat mass, and lean mass were measured pre- and postintervention by DXA using the same Hologic Discovery scanner (Hologic).

### Resting energy metabolism

Pre- and postintervention, energy expenditure and substrate utilization were measured in the overnight fasted state. Gas exchange was measured by open-circuit respirometry with an automated ventilated hood system (Omnical; Maastricht University) for 45 min. Data from the first 10 and last 5 min were omitted. The Weir equation (31) was used to calculate whole-body resting energy expenditure from measurements of oxygen consumption and carbon dioxide production. Carbohydrate and fat oxidation rates were calculated using the nonprotein equations by Péronnet and Massicotte (32).

### Physical performance

In the early afternoon of the first test day, both pre- and postintervention, 2 physical performance tests were performed: a 6-min walk test (6MWT) and a timed chair-stand test (TCST). During the 6MWT, participants were asked to walk as far as possible in 6 min, without running, on a 10-m flat track. For the TCST, participants had to complete 10 chair-rise repetitions as fast as possible, and time was recorded. Preceding each test, a practice round was performed at a slow pace to familiarize with the test.

In the early afternoon of the second test day, both pre- and postintervention, muscle strength, and muscle endurance were measured using the Biodex System 3 Pro dynamometer (Biodex Medical Systems). For both measurements, the participants were stabilized in the chair with shoulder, leg, and abdominal straps to prevent compensatory movement, and the tests were performed with the left leg. To measure maximal muscle strength, each participant performed three 5-s maximal extensions and flexions with a 30-s rest period in between trials. The knee position was fixed at a 70° angle. Maximal isometric knee-extensor and knee-flexor torque was defined as the average of the highest 2 out of 3 peak torques, recorded in Newton-meters. For muscle endurance, participants had to perform 30 consecutive extension and flexion movements (range of motion 120°/s). The peak torque of each extension and flexion was recorded, and a linear trend line was fitted through these peaks. To exclude inconsistent start-up and end performance, the first 5 contractions and the last contraction were omitted before fitting the trend lines. The slope of the trend lines was used as a marker of muscle endurance, where a steeper downward tilt implies a faster decline in muscle strength and hence less muscle endurance, expressed in Newton-meters per contraction.

### Quality of sleep and quality of life

Quality of sleep during the previous month was assessed by the Pittsburgh Sleep Quality Index (PSQI) (33). The PSQI used was a 10-item self-reported questionnaire resulting in a score between 0 and 21, with a lower score indicating a better sleep quality. Quality of life (QOL) was assessed by a 32-item survey (34), with QOL envisioning domains of social, emotional, cognitive, physical, and spiritual well-being all contributing to a combined QOL score. The survey gives a score between 32 and 160 points, with a higher score indicating a better QOL.

### Statistical analysis

Statistics were performed using SPSS version 24.0 for Mac OS, and *P* < 0.05 was considered statistically significant. Preintervention differences between treatment arms were evaluated with a 1-way ANOVA. Postintervention differences between treatment arms in outcome variables were compared using 1-way ANCOVA, implementing the corresponding preintervention variables as covariates. Data are presented as *F*(df_between_, df_within_) = test statistic and/or as the estimated marginal mean (the adjusted mean) and SE. The Shapiro–Wilk normality test was used to evaluate normality distribution. If data were not normally distributed, a generalized linear model was used to test for statistical differences.

Sample size calculation was performed with the change in insulin sensitivity (Matsuda index) as the primary outcome measure of the study. The sample size was calculated with a power of 80% and a significance level of 0.05 using the following formula (*z*_0.8_ = 0.84 and *z*_0.95_ = 1.96):

(1) \begin{eqnarray*} {n_1} = {n_2} = {\rm{ }}\left( {{\sigma _1}^2 + {\sigma _2}^2} \right){\rm{ }} \times {\rm{ }}{\left( {{z_{0.8}} + {z_{0.95}}} \right)^2}/\Delta {{\rm{\mu }}^2} \end{eqnarray*}

Based on previous studies within our research group, the combined intra- and interindividual variability upon 2 repeated OGTTs in Matsuda index is expected to be ∼0.32. Furthermore, we expect a difference of 0.3 on the Matsuda index, using 2 repeated OGTTs, due to resveratrol supplementation (Δμ) (11, 35). Because the expected dropout rate was 15%, 21 participants per treatment arm were recruited.

## Results

### Study compliance

**FIGURE 1:**
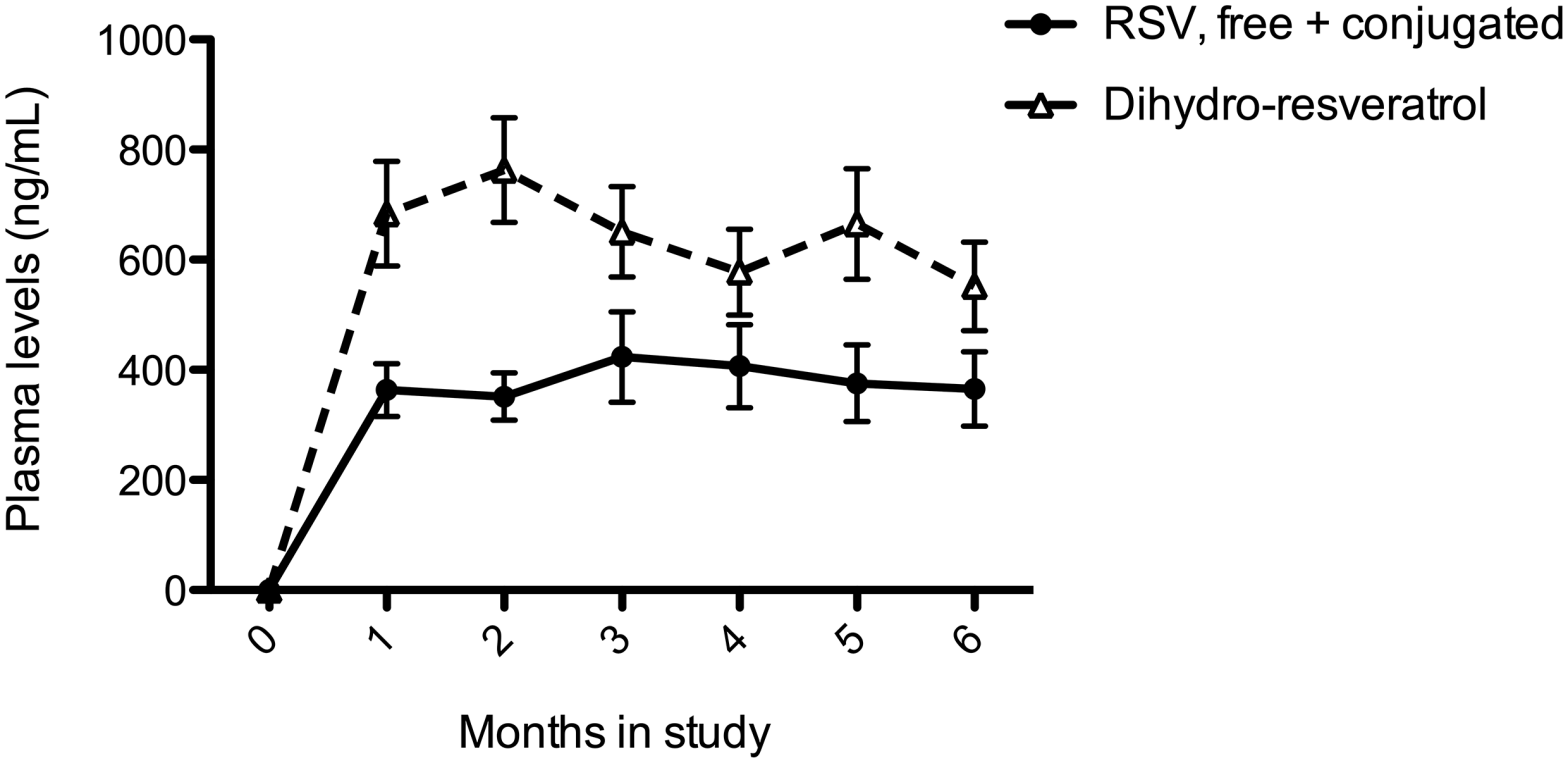
Plasma concentrations of free + conjugated and dihydro-resveratrol of participants in the RSV treatment arm (*n* = 20). Plasma samples were obtained in the overnight fasted state on a monthly basis. Data are presented as means ± SEs. RSV, resveratrol.

Compliance was confirmed by capsule counting and analysis of plasma concentrations of resveratrol (free + conjugated) and dihydro-resveratrol (DHR), a metabolite of resveratrol, on a monthly basis. Resveratrol and DHR concentrations were always below detection in the placebo arm, whereas in the resveratrol arm both were measurable at each monthly visit (**Figure 1**). In the resveratrol arm, mean parental resveratrol concentration was 365 ± 67 ng/mL and mean DHR concentration was 552 ± 81 ng/mL at the end of the 6-mo intervention period, which are in the same range as those in our previous short-term resveratrol studies (14, 23, 36).

### Baseline characteristics

Forty-one participants (24 men, 17 women) with a mean age of 62 ± 1.0 y completed the study. The participants were overweight to obese, with a mean BMI of 29 ± 0.4 (**Table 1**). Preintervention, no differences were observed between the 2 treatment arms for subject characteristics or any of the outcome parameters. No serious adverse events or side effects were observed. During the entire intervention period, participants were encouraged to maintain their initial body weight and physical activity level. Indeed, no differences were observed in physical activity level and total body weight in either of the treatment arms during the 6 mo of intervention (**Supplemental Table 1**). There was no significant difference in postintervention systolic [*F*(1, 35) = 0.112, *P* = 0.740] or diastolic blood pressure [*F*(1, 35) = 0.180, *P* = 0.674] between the 2 treatment arms adjusted for preintervention blood pressure.

**TABLE 1:** Preintervention participant characteristics per treatment arm^1^

	Treatment	
Variable	Resveratrol (*n* = 20)	Placebo (*n* = 21)
Gender, *n* (M/F)	12/8	12/9
Family history of T2D^2^, *n*	6	6
Age, y	61 ± 1.3	62 ± 1.5
Body weight, kg	87.7 ± 2.68	88.3 ± 2.21
BMI, kg/m^2^	30 ± 0.5	29 ± 0.5
Systolic blood pressure, mm Hg	135 ± 3.5	132 ± 2.2
Diastolic blood pressure, mm Hg	88 ± 2.2	83 ± 1.7
Physical activity score^3^	8.0 ± 0.22	7.8 ± 0.26
Medication use, *n*	5	5
Antihypertensives	3	3
Statins	1	2
Other	3	5

1 Values are means ± SEs. T2D, type 2 diabetes.

2 First-degree relative of person with T2D.

3 Total physical activity score assessed by Baecke's habitual physical activity questionnaire.

### Insulin sensitivity

**FIGURE 2:**
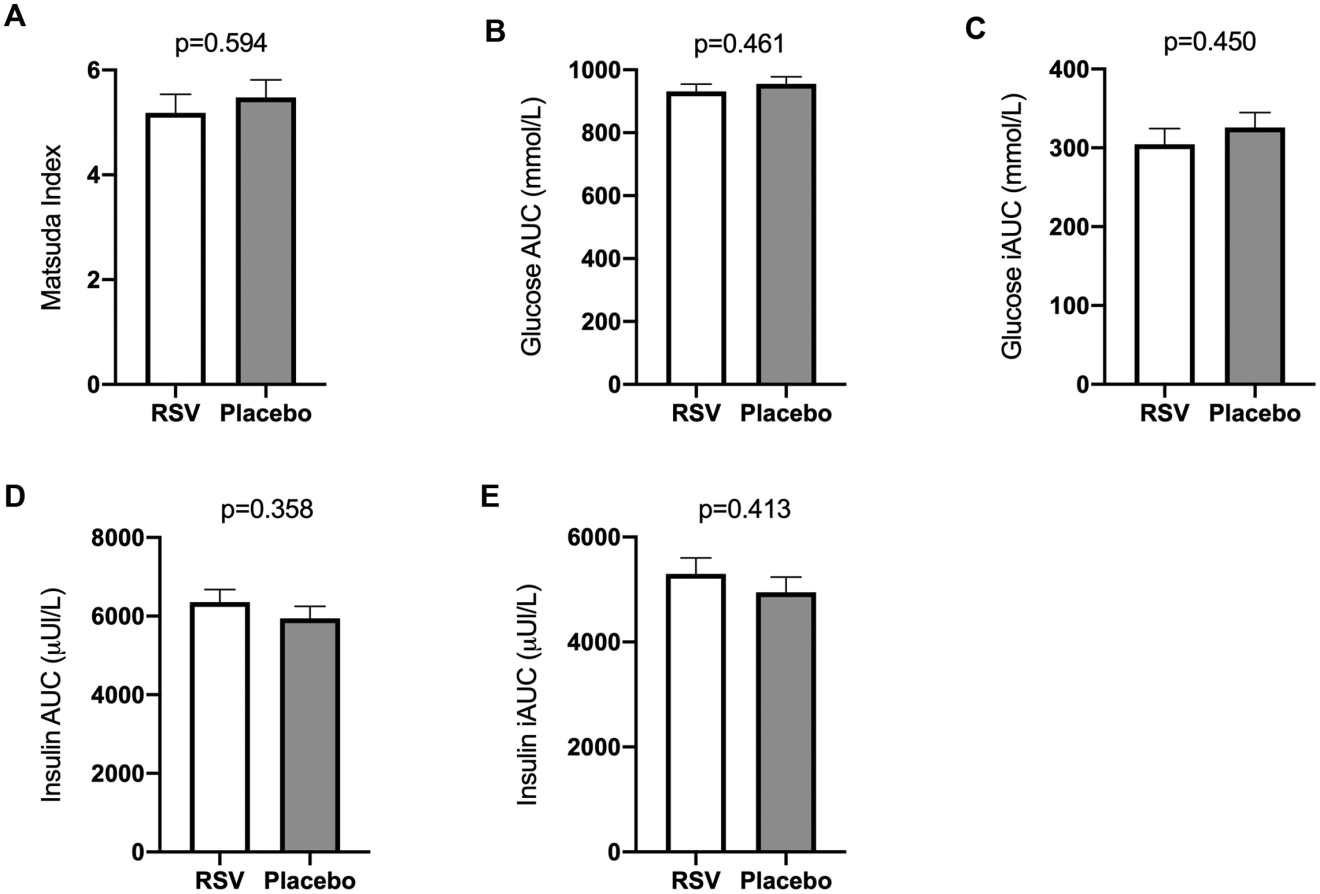
Effects of RSV supplementation on insulin sensitivity. Matsuda index, glucose and insulin AUC, and glucose and insulin iAUC were assessed by data from double 2-h 75-g OGTTs pre- and postintervention. Postintervention differences between treatment arms were compared using 1-way ANCOVA, implementing the corresponding preintervention variables as covariates. Postintervention data are presented as adjusted means ± SEs. Data from 1 participant were omitted because of medication usage interfering with glucose homeostasis shortly before postintervention measurements (*n* = 19 in the RSV arm; *n* = 21 in the placebo arm). (A) Postintervention Matsuda index as estimate for insulin sensitivity. (B–E) Postintervention plasma glucose and insulin iAUC obtained during 2-h OGTTs. Plasma glucose and insulin iAUC were based on 5 and 3 blood sampling points, respectively. iAUC, incremental AUC; OGTT, oral-glucose-tolerance test; RSV, resveratrol.

Postintervention insulin sensitivity, based on the Matsuda index, showed no difference between the resveratrol and placebo treatment arms [*F*(1, 37) = 0.366, *P* = 0.549; **Figure 2**A]. The adjusted mean of the Matsuda index was 5.18 ± 0.35 in the resveratrol arm compared with 5.50 ± 0.34 in the placebo arm. In addition, postintervention glucose AUC and iAUC and insulin AUC and iAUC measured during the OGTTs were also comparable between the arms (Figure 2B–E). Corresponding, no postintervention differences were measured in fasting glucose and insulin, adjusted for preintervention glucose and insulin values (**Table 2**). Postintervention HbA1c was significantly different between the resveratrol and placebo treatment arms [*F*(1, 36) = 8.085, *P* = 0.007], adjusted for preintervention HbA1c. The adjusted means showed that HbA1c was lower on resveratrol (35.8 ± 0.43 mmol/mol) compared with placebo (37.6 ± 0.44 mmol/mol) (Table 2).

**TABLE 2:** Postintervention fasting plasma biochemistry adjusted for baseline variables^1^

	Treatment		
Variable	Resveratrol (*n* = 20)	Placebo (*n* = 21)	*P* value
Glucose, mmol/L	5.2 ± 0.07	5.2 ± 0.06	0.945
Insulin, μU/mL	8.6 ± 0.42	8.3 ± 0.40	0.671
HbA1c, mmol/mol	35.8 ± 0.43	37.6 ± 0.44	0.007
Triglycerides, mmol/L	1.6 ± 0.10	1.7 ± 0.10	0.468
Free fatty acids, µmol/L^2^	531 ± 30	510 ± 29	0.625
Total cholesterol, mmol/L	5.4 ± 0.13	5.5 ± 0.12	0.696
HDL cholesterol, mmol/L	1.4 ± 0.04	1.5 ± 0.04	0.462
LDL cholesterol, mmol/L	3.3 ± 0.11	3.3 ± 0.10	0.778
Bilirubin, µmol/L	11 ± 0.7	11 ± 0.7	0.487
Creatinine, µmol/L^3^	81 ± 2.4	84 ± 2.4	0.249
Urea, mmol/L^4^	5.2 ± 0.20	5.3 ± 0.20	0.511
AST, U/L	24 ± 1.2	23 ± 1.1	0.441
ALT, U/L	26 ± 1.5	25 ± 1.5	0.536

1 Postintervention data are presented as adjusted means ± SEs. Postintervention variables were compared using 1-way ANCOVA, implementing the corresponding baseline variables as covariates. ALT, alanine transaminase; AST, aspartate transaminase; HbA1c, glycated hemoglobin.

2 Data from 1 participant were missing in the resveratrol arm.

3 Data from 1 participant were missing in the placebo arm.

4 Data from 2 participants were missing in the resveratrol arm.

### Plasma biochemistry

In addition to plasma markers related to glucose homeostasis, markers for dyslipidemia and safety were analyzed. Plasma markers related to dyslipidemia remained unaffected by resveratrol treatment (Table 2). In addition, no changes were observed in plasma markers related to safety: bilirubin, creatinine, urea, AST, and ALT did not differ postintervention between the 2 treatment arms, adjusted for their corresponding preintervention values (Table 2).

### IHL content

**FIGURE 3:**
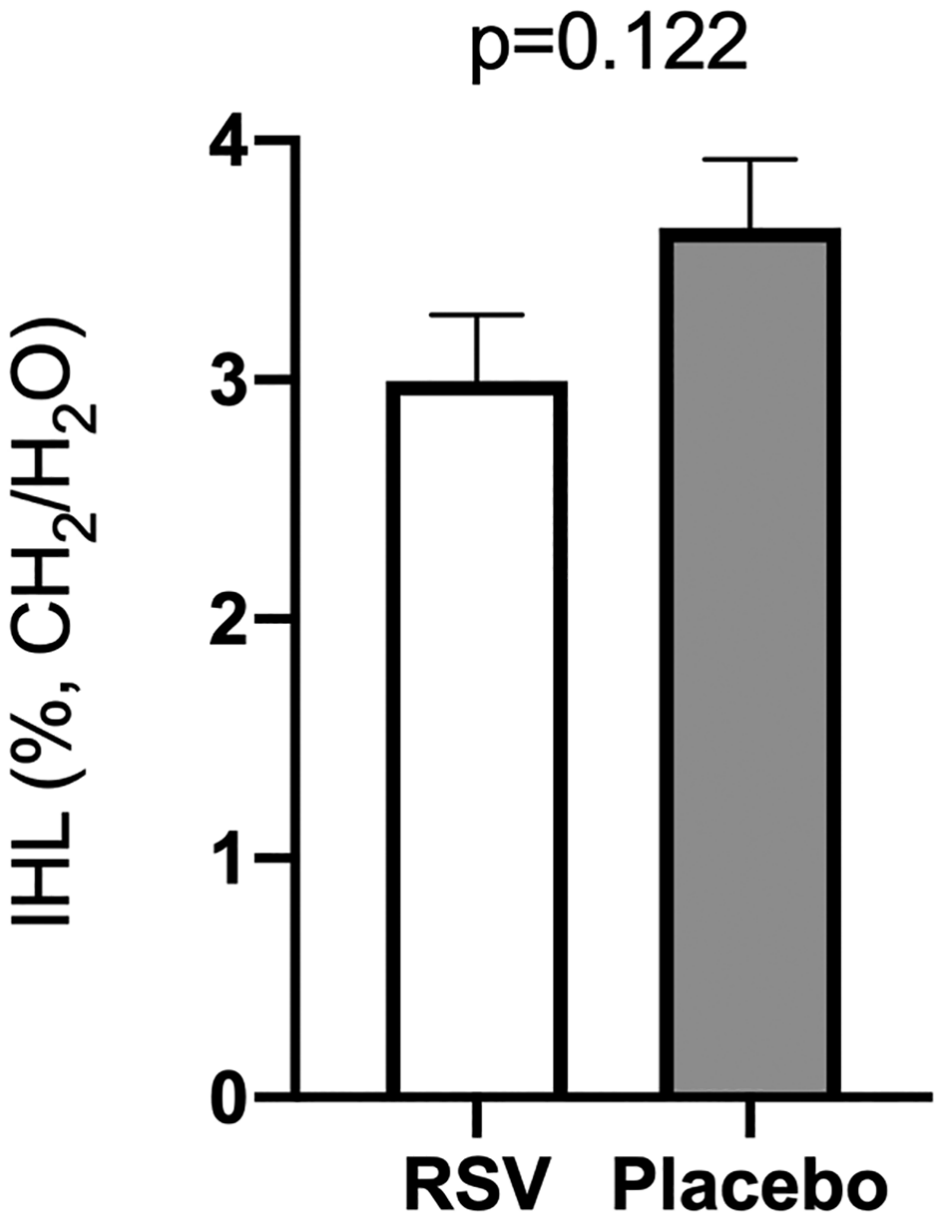
Effect of RSV supplementation on IHL content. IHL content was determined pre- and postintervention by proton magnetic spectroscopy (^1^H-MRS) on a 3-T MRI scanner. Data are given as T2 corrected ratios of the CH_2_ peak relative to unsuppressed water resonance, expressed as percentage. Postintervention differences between treatment arms were compared with 1-way ANCOVA, implementing the corresponding preintervention variables as covariates. Postintervention data are presented as adjusted means ± SEs. Data from 2 participants were missing due to claustrophobia, and data from 2 measurements were omitted because of motion artifacts (*n* = 19 in the RSV arm; *n* = 18 in the placebo arm). IHL, intrahepatic lipid; RSV, resveratrol.

IHL content data pre- and postintervention were not normally distributed. However, the residuals were normally distributed and hence assumptions for ANCOVA were met. There was no significant difference in postintervention IHL content between the treatment arms, corrected for preintervention IHL content [*F*(1, 34) = 2.510, *P* = 0.122; **Figure 3**].

### Body composition

**FIGURE 4:**
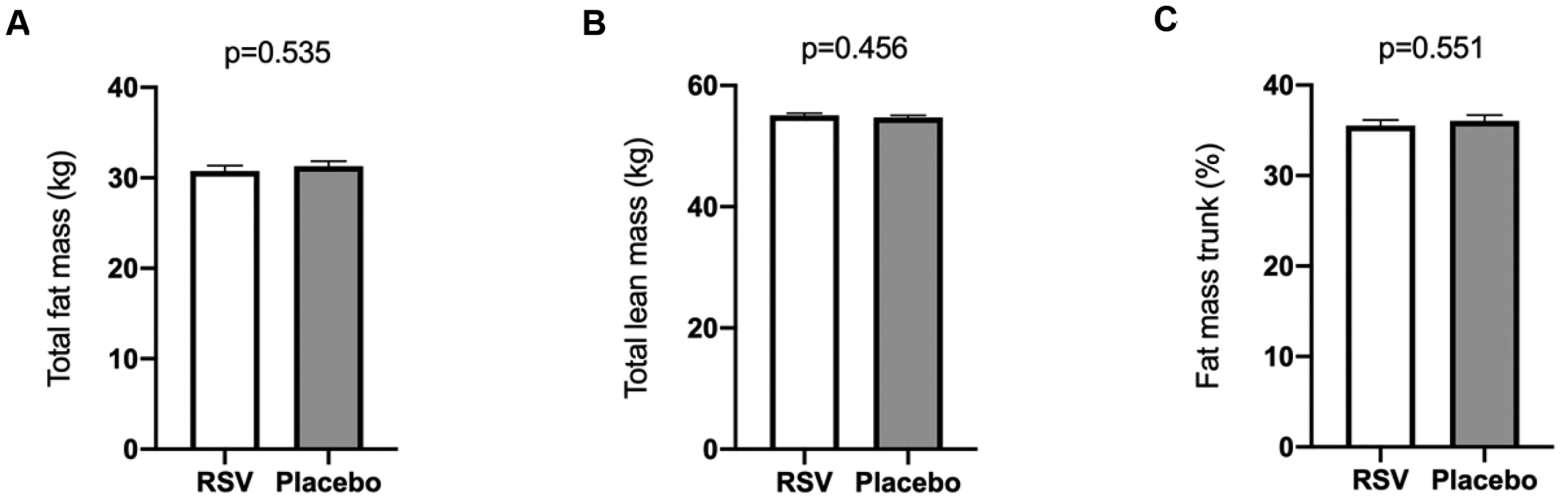
Effects of RSV supplementation on body composition. Body composition was determined pre- and postintervention using DXA. Postintervention differences between treatment arms were compared using 1-way ANCOVA, implementing the corresponding preintervention variables as covariates. Postintervention data are presented as adjusted means ± SEs, *n* = 20 in the RSV arm and *n* = 21 in the placebo arm. RSV, resveratrol.

As described previously, no difference was observed in total body weight between the treatment arms. There was also no significant difference in postintervention total fat mass [*F*(1, 38) = 0.392, *P* = 0.535; **Figure 4**A] or total lean mass [*F*(1, 38) = 0.567, *P* = 0.456; Figure 4B] between the treatment arms, adjusted for preintervention fat and lean mass, respectively. Also, when specifically examining fat percentage of the trunk, no significant difference was observed between the 2 arms [*F*(1, 38) = 0.362, *P* = 0.551; Figure 4C].

### Resting energy metabolism

Previously, it has been shown that resveratrol can lower sleeping metabolic rate and the respiratory exchange ratio. Nevertheless, in this study no differences were found between the 2 treatment arms in postintervention resting energy expenditure, respiratory exchange ratio, carbohydrate oxidation, and fat oxidation, adjusted for corresponding preintervention data (**Table 3**).

**TABLE 3:** Postintervention resting energy metabolism adjusted for baseline^1^

	Treatment		
Variable	Resveratrol (*n* = 20)	Placebo (*n* = 21)	*P* value
Energy expenditure, kJ/min	4.91 ± 0.06	4.92 ± 0.06	0.917
Carbohydrate oxidation, g/min	0.10 ± 0.01	0.10 ± 0.01	0.948
Fat oxidation, g/min	0.08 ± 0.00	0.08 ± 0.00	0.972
Respiratory exchange ratio	0.79 ± 0.01	0.80 ± 0.01	0.758

1 Values are adjusted means ± SEs. Postintervention variables were compared using 1-way ANCOVA, implementing the corresponding baseline variables as covariates.

### Physical performance

**FIGURE 5:**
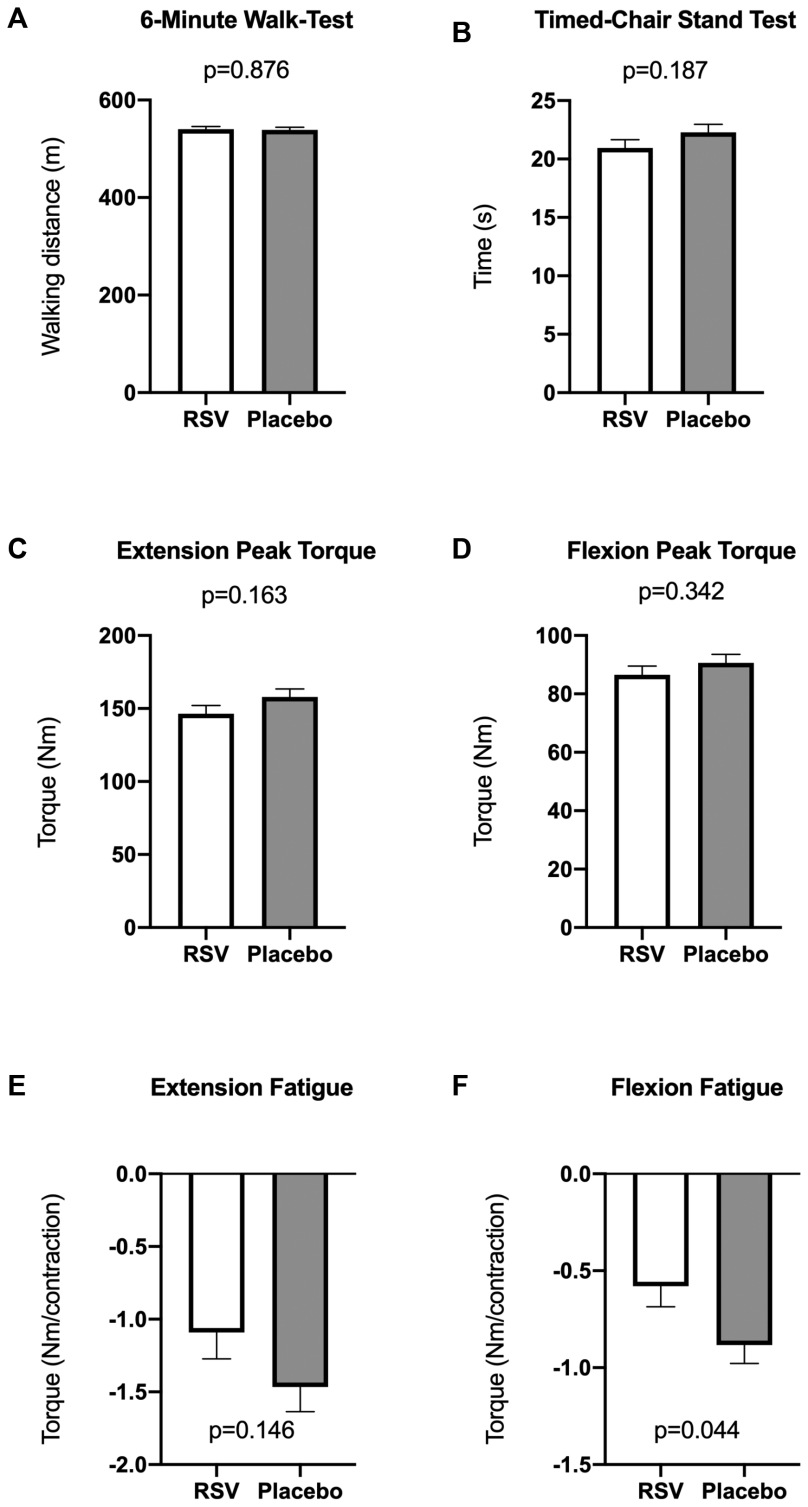
Effects of RSV on functional parameters of physical performance. Physical performance tests were performed pre- and postintervention. Postintervention differences between treatment arms were compared using 1-way ANCOVA, implementing the corresponding preintervention variables as covariates. Postintervention data are presented as adjusted means ± SEs. (A) Post-treatment distance covered during the 6-min walk test per treatment arm. Data from 5 participants were omitted because of pain in the ankle, hip, or knee (*n* = 17 in the RSV arm; *n* = 19 in the placebo arm). (B) Postintervention time to complete the timed chair-stand test. Data from 2 participants were omitted because of pain in the ankle or hip (*n* = 19 in the RSV arm; *n* = 20 in the placebo arm). (C, D) Postintervention maximal isometric muscle extension and flexion strength measured by the Biodex system by isometric test. Data from 4 participants were not obtained because of technical problems (*n* = 18 in the RSV arm; *n* = 19 in the placebo arm). (E, F) Postintervention muscle isokinetic extension and flexion endurance, expressed as the slope of the trend lines measured by the Biodex system by isokinetic test. Data from 4 participants were not included because of technical problems, and those from 10 participants were not included because of no decline in muscle endurance over the 30 repetitions (*n* = 12 in the RSV arm; *n* = 14 in the placebo arm). RSV, resveratrol.

Walking distance during the 6MWT increased significantly within both treatment arms (resveratrol: Δ15.6 ± 5.99 m; placebo: Δ14.5 ± 5.60 m), with no postintervention differences between the arms [*F*(1, 33) = 0.025, *P* = 0.876; **Figure 5**A]. Postintervention stand–sit time, measured during the TCST, was not significantly different between the placebo and resveratrol arms [*F*(1, 36) = 1.813, *P* = 0.187; Figure 5B]. There was no difference in maximal muscle strength for both extension and flexion movement as represented by peak torque during the Biodex exercises (Figure 5C, D). Similarly, there was no postintervention difference between the treatment arms in muscle endurance for the extension movement (Figure 5E). However, there was a significant difference in postintervention endurance for the muscle flexion movement [*F*(1, 24) = 4.522, *P* = 0.044; Figure 5F]. The adjusted means showed that flexion strength decreased slower on resveratrol (0.58 ± 0.11 Nm/contraction) compared with placebo (0.88 ± 0.09 Nm/contraction), representing an improved muscle endurance.

### Quality of sleep and quality of life

There were no differences in postintervention scores for quality of sleep [*F*(1, 38) = 0.832, *P* = 0.367] and quality of life [*F*(1, 38) = 0.303, *P* = 0.585] between resveratrol and placebo treatment arms as assessed by questionnaires, adjusted for preintervention scores.

## Discussion

In this randomized, placebo-controlled, parallel-group clinical trial, we investigated the effects of long-term treatment with 150 mg resveratrol per day in an overweight population of men and women on metabolic health. In this study, resveratrol supplementation did not result in a higher insulin sensitivity as assessed by the Matsuda index, compared with placebo. Also, other markers related to insulin sensitivity, such as fasting plasma glucose and insulin and glucose and insulin during the OGTT, were unaffected except for HbA1c. Postintervention HbA1c was significantly lower in the resveratrol treatment arm compared with the placebo arm. HbA1c is a marker of long-term glycemic control, and it reflects average plasma glucose values of the preceding 3 mo (37). This beneficial effect of resveratrol on HbA1c has been found previously, but mainly in patients with T2D, as described in a meta-analysis by Liu et al. (38). A study by Witte et al. (39) found comparable results when 200 mg/d resveratrol was supplied for 6 mo. Witte et al. also found a significant reduction in HbA1c but no improvement in fasting glucose and even an increase in fasting insulin concentrations upon resveratrol supplementation (39). Observing an effect on HbA1c and not on plasma glucose concentrations could imply an alteration in the glycation of hemoglobin or erythrocyte life span rather than a decrease in average plasma glucose concentrations. Indeed, previous studies using cell models have indicated that resveratrol can interact with hemoglobin and influence erythrocyte metabolism (40, 41). However, it is unknown if this interaction can lead to alterations in HbA1c. In addition, clinical trials that did find improvements in glucose hemostasis or insulin sensitivity generally used a higher dose of resveratrol, ranging from 480 to 2000 mg/d (11–13, 42).

In addition to plasma markers related to glucose homeostasis, we examined markers related to dyslipidemia. We found that none of these plasma markers were affected by resveratrol treatment: total cholesterol, HDL and LDL cholesterol, free fatty acids, and triglycerides remained unaffected during the 6-mo intervention period. To date, a few human clinical trials have found reductions in cholesterol, whereas most studies have reported inconclusive results or have not found effects (43, 44). A study by Kjaer et al. (45) even reported an increase in total and LDL cholesterol upon treatment with 1000 mg resveratrol per day for 16 wk. The combination of the results of our clinical trial and those of previous clinical trials indicates that resveratrol appears unsuitable for treating dyslipidemia. Finally, we measured plasma markers related to safety: bilirubin, creatinine, urea, AST, and ALT. None of the plasma markers changed during the 6 mo, neither within or between the groups, which implies that supplying 150 mg of resveratrol per day for 6 mo can be regarded as safe, as also previously established (45).

We did not find statistically significant effects of resveratrol supplementation on IHL content, despite the fact that our group previously established that the same dose of resveratrol did decrease liver fat content in a 30-d clinical trial in healthy obese men (8). First, a lack of power may be responsible for the lack of statistically significant lower IHL concentrations. Furthermore, the current study used a more mixed population, including participants with impaired glucose tolerance and both men and women. Other clinical trials in which participants were enrolled with disturbed glucose homeostasis, such as patients with T2D, the metabolic syndrome, or decreased insulin sensitivity, also did not find improvement in IHL upon resveratrol treatment (14, 36, 45, 46). This may suggest that resveratrol only lowers IHL in healthy overweight individuals and that other factors may prevent improvements in IHL in metabolically compromised individuals.

Animal studies suggest that resveratrol could protect against high-fat-diet-induced weight gain and stimulate energy expenditure. To date, no effects of resveratrol on body weight or body composition have been established in humans (8, 9, 11, 12, 15, 17). In accordance, we found no significant effects of resveratrol on body composition compared with placebo. We also did not find any changes in resting metabolic rate in either of the groups. Resting energy expenditure and fat and carbohydrate utilization remained stable during the 6-mo intervention period. This is in contradiction to animal studies (47–49), but it is in agreement with most human clinical trials in which no effects of resveratrol treatment have been observed on resting energy metabolism (7, 14).

Skeletal muscle plays an important role in glucose metabolism, and impaired skeletal muscle functioning contributes to the development of T2D (50). Because the mitochondria produce >90% of the ATP needed for movement, improvements in mitochondrial function can lead to improved muscle functioning. It is known that resveratrol can induce the activity of peroxisome proliferator–activated receptor γ coactivator 1α in mice (47), a key regulator of mitochondrial biogenesis (5). Studies in rodents indicate that resveratrol supplementation combined with exercise training can improve muscle strength and endurance more than exercise training alone (51). Multiple human clinical trials have also revealed that a relatively low dose of resveratrol supplementation can improve *ex vivo* muscle mitochondrial oxidative capacity after 30 d (8, 14, 22, 36). Whether improvements in mitochondrial oxidative capacity are also reflected in improved muscle function has only been investigated in 2 human clinical trials with mixed results (24, 25). Therefore, in this clinical trial, we focused on the potential effects of resveratrol on muscle strength and endurance. To this end, the participants performed several physical performance tests: a 6MWT, a TCST, and a knee extensor strength and endurance test. Distance covered during 6 min of walking was not different between the resveratrol arm and the placebo arm. A recent clinical trial also did not find effects of resveratrol on walking performance (25). In addition, in our clinical trial, no effects were seen on time to complete 10 chair-stand movements and muscle strength and endurance measured by the Biodex System when the resveratrol arm was compared with the placebo arm. Only a small beneficial effect of resveratrol on endurance of the flexion movement was found compared with placebo. Finally, resveratrol did not improve quality of life or quality of sleep, assessed by questionnaires.

### Strengths and limitations

The OGTT was used to measure the effect of resveratrol on the primary outcome measure insulin sensitivity. OGTTs have a higher within-subject variability than that of the gold standard hyperinsulinemic euglycemic clamp technique and are therefore less reliable for measuring insulin sensitivity. To overcome this, the OGTT was performed twice at the beginning and twice at the end of the intervention period, thereby reducing interindividual variability and improving reliability. Furthermore, the study was not powered to measure sex differences. Because only postmenopausal women were included, gender effects are expected to be minimal. In addition, no multiple testing correction was applied, increasing the risk of false positives. The main strengths of this study are the randomized, double-blind, placebo-controlled study design; the relatively long treatment period of 6 mo; use of validated methods; high compliance to treatment; and low dropout rate.

### Conclusions

Resveratrol 150 mg/d for 6 mo had no beneficial effect on insulin sensitivity, assessed by the Matsuda index, nor on outcome parameters related to liver fat accumulation, body composition, dyslipidemia, energy metabolism, physical performance, and quality of life and sleep, compared with placebo. Resveratrol supplementation did give lower HbA1c concentrations compared with the placebo arm. Supplying resveratrol with a higher dose may be needed to achieve more profound health effects.

## Supplementary Material

nqaa125_Supplemental_File

## Notes

This study was funded by Diabetes Fonds project grant 2012.00.1525 and the Netherlands Cardiovascular Research Initiative: an initiative with support of the Dutch Heart Foundation (CVON2014-02ENERGISE). DSM Nutritional Products provided the resveratrol and placebo capsules.

Supplemental Figures 1 and 2 and Supplemental Table 1 are available from the “Supplementary data” link in the online posting of the article and from the same link in the online table of contents at https://academic.oup.com/ajcn/.

Data described in the manuscript, code book, and analytic code will be made available upon request pending approval.

Abbreviations used: ALT, alanine aminotransferase; AST, aspartate aminotransferase; DHR, dihydro-resveratrol; HbA1c, glycated hemoglobin; iAUC, incremental AUC; IHL, intrahepatic lipid; MRS, magnetic resonance spectroscopy; OGTT, oral-glucose-tolerance test; PSQI, Pittsburgh Sleep Quality Index; QOL, quality of life; SIRT1, sirtuin 1; TCST, timed chair-stand test; T2D, type 2 diabetes mellitus; 6MWT, 6-min walk test.

## References

[1] WHO. Fact sheet: obesity and overweight. Geneva (Switzerland): WHO;2017.

[2] Dube JJ, Amati F, Toledo FG, Stefanovic-Racic M, Rossi A, Coen P, Goodpaster BH Effects of weight loss and exercise on insulin resistance, and intramyocellular triacylglycerol, diacylglycerol and ceramide. Diabetologia. 2011;54(5):1147–56. 21327867 10.1007/s00125-011-2065-0 PMC3804898

[3] Cruzen C, Colman RJ. Effects of caloric restriction on cardiovascular aging in non-human primates and humans. Clin Geriatr Med. 2009;25(4):733. 19944270 10.1016/j.cger.2009.07.001 PMC2786902

[4] Middleton KR, Anton SD, Perri MG Long-term adherence to health behavior change. Am J Lifestyle Med. 2013;7(6):395–404. 27547170 10.1177/1559827613488867 PMC4988401

[5] Howitz KT, Bitterman KJ, Cohen HY, Lamming DW, Lavu S, Wood JG, Zipkin RE, Chung P, Kisielewski A, Zhang LL et al. Small molecule activators of sirtuins extend *Saccharomyces cerevisiae* lifespan. Nature. 2003;425(6954):191–6. 12939617 10.1038/nature01960

[6] Yu J, Auwerx J The role of sirtuins in the control of metabolic homeostasis. Ann N Y Acad Sci. 2009;1173(Suppl 1):E10–9. 19751409 10.1111/j.1749-6632.2009.04952.x PMC3620552

[7] de Ligt M, Timmers S, Schrauwen P Resveratrol and obesity: can resveratrol relieve metabolic disturbances?. Biochim Biophys Acta. 2015;1852(6):1137–44. 25446988 10.1016/j.bbadis.2014.11.012

[8] Timmers S, Konings E, Bilet L, Houtkooper RH, van de Weijer T, Goossens GH, Hoeks J, van der Krieken S, Ryu D, Kersten S et al. Calorie restriction-like effects of 30 days of resveratrol supplementation on energy metabolism and metabolic profile in obese humans. Cell Metab. 2011;14(5):612–22. 22055504 10.1016/j.cmet.2011.10.002 PMC3880862

[9] Bhatt JK, Thomas S, Nanjan MJ Resveratrol supplementation improves glycemic control in type 2 diabetes mellitus. Nutr Res. 2012;32(7):537–41. 22901562 10.1016/j.nutres.2012.06.003

[10] Brasnyo P, Molnar GA, Mohas M, Marko L, Laczy B, Cseh J, Mikolas E, Szijarto IA, Merei A, Halmai R et al. Resveratrol improves insulin sensitivity, reduces oxidative stress and activates the Akt pathway in type 2 diabetic patients. Br J Nutr. 2011;106(3):383–9. 21385509 10.1017/S0007114511000316

[11] Crandall JP, Oram V, Trandafirescu G, Reid M, Kishore P, Hawkins M, Cohen HW, Barzilai N Pilot study of resveratrol in older adults with impaired glucose tolerance. J Gerontol Ser A Biol Sci Med Sci. 2012;67(12):1307–12. 22219517 10.1093/gerona/glr235 PMC3670158

[12] Movahed A, Nabipour I, Lieben Louis X, Thandapilly SJ, Yu L, Kalantarhormozi M, Rekabpour SJ, Netticadan T Antihyperglycemic effects of short term resveratrol supplementation in type 2 diabetic patients. Evid Based Complement Alternat Med. 2013;2013:851267. 24073011 10.1155/2013/851267 PMC3773903

[13] Mendez-del Villar M, Gonzalez-Ortiz M, Martinez-Abundis E, Perez-Rubio KG, Lizarraga-Valdez R Effect of resveratrol administration on metabolic syndrome, insulin sensitivity, and insulin secretion. Metab Syndr Relat Disord. 2014;12(10):497–501. 25137036 10.1089/met.2014.0082

[14] Timmers S, de Ligt M, Phielix E, van de Weijer T, Hansen J, Moonen-Kornips E, Schaart G, Kunz I, Hesselink MK, Schrauwen-Hinderling VB et al. Resveratrol as add-on therapy in subjects with well-controlled type 2 diabetes: a randomized controlled trial. Diabetes Care. 2016;39(12):2211–7. 27852684 10.2337/dc16-0499

[15] Yoshino J, Conte C, Fontana L, Mittendorfer B, Imai S, Schechtman KB, Gu C, Kunz I, Rossi Fanelli F, Patterson BW et al. Resveratrol supplementation does not improve metabolic function in nonobese women with normal glucose tolerance. Cell Metab. 2012;16(5):658–64. 23102619 10.1016/j.cmet.2012.09.015 PMC3496026

[16] Dash S, Xiao C, Morgantini C, Szeto L, Lewis GF High-dose resveratrol treatment for 2 weeks inhibits intestinal and hepatic lipoprotein production in overweight/obese men. Arterioscler Thromb Vasc Biol. 2013;33(12):2895–901. 24072699 10.1161/ATVBAHA.113.302342

[17] Poulsen MM, Vestergaard PF, Clasen BF, Radko Y, Christensen LP, Stodkilde-Jorgensen H, Moller N, Jessen N, Pedersen SB, Jorgensen JO High-dose resveratrol supplementation in obese men: an investigator-initiated, randomized, placebo-controlled clinical trial of substrate metabolism, insulin sensitivity, and body composition. Diabetes. 2013;62(4):1186–95. 23193181 10.2337/db12-0975 PMC3609591

[18] Chachay VS, Macdonald GA, Martin JH, Whitehead JP, O'Moore-Sullivan TM, Lee P, Franklin M, Klein K, Taylor PJ, Ferguson M et al. Resveratrol does not benefit patients with nonalcoholic fatty liver disease. Clin Gastroenterol Hepatol. 2014;12(12):2092–103. 24582567 10.1016/j.cgh.2014.02.024

[19] Olesen J, Gliemann L, Bienso R, Schmidt J, Hellsten Y, Pilegaard H Exercise training, but not resveratrol, improves metabolic and inflammatory status in skeletal muscle of aged men. J Physiol. 2014;592(8):1873–86. 24514907 10.1113/jphysiol.2013.270256 PMC4001758

[20] van der Made SM, Plat J, Mensink RP Resveratrol does not influence metabolic risk markers related to cardiovascular health in overweight and slightly obese subjects: a randomized, placebo-controlled crossover trial. PLoS One. 2015;10(3):e0118393. 25790328 10.1371/journal.pone.0118393 PMC4366169

[21] Zortea K, Franco VC, Francesconi LP, Cereser KM, Lobato MI, Belmonte-de-Abreu PS Resveratrol supplementation in schizophrenia patients: a randomized clinical trial evaluating serum glucose and cardiovascular risk factors. Nutrients. 2016;8(2):73. 26840331 10.3390/nu8020073 PMC4772037

[22] Most J, Timmers S, Warnke I, Jocken JW, van Boekschoten M, de Groot P, Bendik I, Schrauwen P, Goossens GH, Blaak EE Combined epigallocatechin-3-gallate and resveratrol supplementation for 12 wk increases mitochondrial capacity and fat oxidation, but not insulin sensitivity, in obese humans: a randomized controlled trial. Am J Clin Nutr. 2016;104(1):215–27. 27194304 10.3945/ajcn.115.122937

[23] de Ligt M, Bruls YMH, Hansen J, Habets MF, Havekes B, Nascimento EBM, Moonen-Kornips E, Schaart G, Schrauwen-Hinderling VB, van Marken Lichtenbelt W et al. Resveratrol improves *ex vivo* mitochondrial function but does not affect insulin sensitivity or brown adipose tissue in first degree relatives of patients with type 2 diabetes. Mol Metab. 2018;12:39–47. 29706321 10.1016/j.molmet.2018.04.004 PMC6001939

[24] Alway SE, McCrory JL, Kearcher K, Vickers A, Frear B, Gilleland DL, Bonner DE, Thomas JM, Donley DA, Lively MW et al. Resveratrol enhances exercise-induced cellular and functional adaptations of skeletal muscle in older men and women. J Gerontol A Biol Sci Med Sci. 2017;72(12):1595–606. 28505227 10.1093/gerona/glx089 PMC5861947

[25] McDermott MM, Leeuwenburgh C, Guralnik JM, Tian L, Sufit R, Zhao L, Criqui MH, Kibbe MR, Stein JH, Lloyd-Jones D et al. Effect of resveratrol on walking performance in older people with peripheral artery disease: the RESTORE randomized clinical trial. JAMA Cardiol. 2017;2(8):902–7. 28403379 10.1001/jamacardio.2017.0538 PMC5815080

[26] Baecke JA, Burema J, Frijters JE A short questionnaire for the measurement of habitual physical activity in epidemiological studies. Am J Clin Nutr. 1982;36(5):936–42. 7137077 10.1093/ajcn/36.5.936

[27] Matsuda M, DeFronzo RA. Insulin sensitivity indices obtained from oral glucose tolerance testing: comparison with the euglycemic insulin clamp. Diabetes Care. 1999;22(9):1462–70. 10480510 10.2337/diacare.22.9.1462

[28] Brouwers B, Schrauwen-Hinderling VB, Jelenik T, Gemmink A, Sparks LM, Havekes B, Bruls Y, Dahlmans D, Roden M, Hesselink MKC et al. Exercise training reduces intrahepatic lipid content in people with and people without nonalcoholic fatty liver. Am J Physiol Endocrinol Metab. 2018;314(2):E165–-73. 29118014 10.1152/ajpendo.00266.2017

[29] Guiu B, Petit JM, Loffroy R, Ben Salem D, Aho S, Masson D, Hillon P, Krause D, Cercueil JP Quantification of liver fat content: comparison of triple-echo chemical shift gradient-echo imaging and in vivo proton MR spectroscopy. Radiology. 2009;250(1):95–102. 19092092 10.1148/radiol.2493080217

[30] Lindeboom L, Nabuurs CI, Hesselink MK, Wildberger JE, Schrauwen P, Schrauwen-Hinderling VB Proton magnetic resonance spectroscopy reveals increased hepatic lipid content after a single high-fat meal with no additional modulation by added protein. Am J Clin Nutr. 2015;101(1):65–71. 25527751 10.3945/ajcn.114.094730

[31] Weir JB New methods for calculating metabolic rate with special reference to protein metabolism. J Physiol. 1949;109(1–2):1–9. 15394301 10.1113/jphysiol.1949.sp004363 PMC1392602

[32] Peronnet F, Massicotte D Table of nonprotein respiratory quotient: an update. Can J Sport Sci. 1991;16(1):23–9. 1645211

[33] Buysse DJ, Reynolds CF 3rd, Monk TH, Berman SR, Kupfer DJ The Pittsburgh Sleep Quality Index: a new instrument for psychiatric practice and research. Psychiatry Res. 1989;28(2):193–213. 2748771 10.1016/0165-1781(89)90047-4

[34] Gill DL, Chang YK, Murphy KM, Speed KM, Hammond CC, Rodriguez EA, Lyu M, Shang YT Quality of life assessment for physical activity and health promotion. Appl Res Qual Life. 2011;6(2):181–200.

[35] de Bock M, Derraik JG, Brennan CM, Biggs JB, Morgan PE, Hodgkinson SC, Hofman PL, Cutfield WS Olive (*Olea europaea* L.) leaf polyphenols improve insulin sensitivity in middle-aged overweight men: a randomized, placebo-controlled, crossover trial. PLoS One. 2013;8(3):e57622. 23516412 10.1371/journal.pone.0057622 PMC3596374

[36] de Ligt M, Bruls YMH, Hansen J, Habets M, Havekes B, Nascimento EBM, Moonen-Kornips E, Schaart G, Schrauwen-Hinderling VB, van Marken Lichtenbelt WD et al. Resveratrol improves mitochondrial function but does not affect insulin sensitivity or brown adipose tissue in first degree relatives of patients with type 2 diabetes. Mol Metab. 2018;12:39–47. 29706321 10.1016/j.molmet.2018.04.004 PMC6001939

[37] Colagiuri S Glycated haemoglobin (HbA1c) for the diagnosis of diabetes mellitus—practical implications. Diabetes Res Clin Pract. 2011;93(3):312–3. 21820751 10.1016/j.diabres.2011.06.025

[38] Liu K, Zhou R, Wang B, Mi MT Effect of resveratrol on glucose control and insulin sensitivity: a meta-analysis of 11 randomized controlled trials. Am J Clin Nutr. 2014;99(6):1510–9. 24695890 10.3945/ajcn.113.082024

[39] Witte AV, Kerti L, Margulies DS, Floel A Effects of resveratrol on memory performance, hippocampal functional connectivity, and glucose metabolism in healthy older adults. J Neurosci. 2014;34(23):7862–70. 24899709 10.1523/JNEUROSCI.0385-14.2014 PMC6608268

[40] Galtieri A, Tellone E, Ficarra S, Russo A, Bellocco E, Barreca D, Scatena R, Lagana G, Leuzzi U, Giardina B Resveratrol treatment induces redox stress in red blood cells: a possible role of caspase 3 in metabolism and anion transport. Biol Chem. 2010;391(9):1057–65. 20536388 10.1515/BC.2010.100

[41] Tellone E, De Rosa MC, Pirolli D, Russo A, Giardina B, Galtieri A, Ficarra S Molecular interactions of hemoglobin with resveratrol: potential protective antioxidant role and metabolic adaptations of the erythrocyte. Biol Chem. 2014;395(3):347–54. 24150206 10.1515/hsz-2013-0257

[42] Chen S, Zhao X, Ran L, Wan J, Wang X, Qin Y, Shu F, Gao Y, Yuan L, Zhang Q et al. Resveratrol improves insulin resistance, glucose and lipid metabolism in patients with non-alcoholic fatty liver disease: a randomized controlled trial. Dig Liver Dis. 2015;47(3):226–32. 25577300 10.1016/j.dld.2014.11.015

[43] Haghighatdoost F, Hariri M. Effect of resveratrol on lipid profile: an updated systematic review and meta-analysis on randomized clinical trials. Pharmacol Res. 2018;129:141–50. 29305228 10.1016/j.phrs.2017.12.033

[44] Sahebkar A. Effects of resveratrol supplementation on plasma lipids: a systematic review and meta-analysis of randomized controlled trials. Nutr Rev. 2013;71(12):822–35. 24111838 10.1111/nure.12081

[45] Kjaer TN, Ornstrup MJ, Poulsen MM, Stodkilde-Jorgensen H, Jessen N, Jorgensen JOL, Richelsen B, Pedersen SB No beneficial effects of resveratrol on the metabolic syndrome: a randomized placebo-controlled clinical trial. J Clin Endocrinol Metab. 2017;102(5):1642–51. 28182820 10.1210/jc.2016-2160

[46] Kantartzis K, Fritsche L, Bombrich M, Machann J, Schick F, Staiger H, Kunz I, Schoop R, Lehn-Stefan A, Heni M et al. Effects of resveratrol supplementation on liver fat content in overweight and insulin-resistant subjects: a randomized, double-blind, placebo-controlled clinical trial. Diabetes Obes Metab. 2018;20(7):1793–7. 29484808 10.1111/dom.13268

[47] Lagouge M, Argmann C, Gerhart-Hines Z, Meziane H, Lerin C, Daussin F, Messadeq N, Milne J, Lambert P, Elliott P et al. Resveratrol improves mitochondrial function and protects against metabolic disease by activating SIRT1 and PGC-1α. Cell. 2006;127(6):1109–22. 17112576 10.1016/j.cell.2006.11.013

[48] Dal-Pan A, Blanc S, Aujard F Resveratrol suppresses body mass gain in a seasonal non-human primate model of obesity. BMC Physiol. 2010;10:11. 20569453 10.1186/1472-6793-10-11 PMC2903570

[49] Dal-Pan A, Terrien J, Pifferi F, Botalla R, Hardy I, Marchal J, Zahariev A, Chery I, Zizzari P, Perret M et al. Caloric restriction or resveratrol supplementation and ageing in a non-human primate: first-year outcome of the RESTRIKAL study in *Microcebus murinus*. Age. 2011;33(1):15–31. 20532988 10.1007/s11357-010-9156-6 PMC3063642

[50] DeFronzo RA, Tripathy D. Skeletal muscle insulin resistance is the primary defect in type 2 diabetes. Diabetes Care. 2009;32(Suppl 2):S157–63. 19875544 10.2337/dc09-S302 PMC2811436

[51] Wicinski M, Leis K, Szyperski P, Weclewicz MM, Mazur E, Pawlak-Osinska K Impact of resveratrol on exercise performance: a review. Sci Sport. 2018;33(4):207–12.

