# Supplementary material for: No effect of resveratrol supplementation after 6 months on insulin sensitivity in overweight adults: a randomized trial
Source: Am J Clin Nutr. 2020 Jun 3;112(4):1029–38. doi: 10.1093/ajcn/nqaa125 (PMC7528554; doi:10.1093/ajcn/nqaa125)
Supplement: nqaa125_Supplemental_File [file nqaa125_supplemental_file.docx]

No effect of resveratrol supplementation after 6 months on insulin sensitivity in overweight adults: a randomized trial

M. de Ligt et al.; On-line Supplementary Material

**ON-LINE SUPPLEMENTARY MATERIAL**

No effect of resveratrol supplementation after 6 months on insulin sensitivity in overweight adults: a randomized trial

M. de Ligt et al.; On-line Supplementary Material

Analysed (n=21)
♦ Excluded from analysis (n=0)

Lost to follow-up (n=0)

Discontinued intervention (n=0)

Analysed (n=20)
♦ Excluded from analysis (n=0)

## Analysis

## Follow-Up

Lost to follow-up (n=0)

Discontinued intervention (n=1); change in medication (n=1)

Allocated to placebo (n=21)

♦ Received allocated intervention (n=21)

Allocated to resveratrol (n=21)

♦ Received allocated intervention (n=21)

Excluded (n=22)

♦  Not meeting inclusion criteria (n=19)

♦  Declined to participate (n=3)

## Enrollment

## Allocation

Randomized (n=42)

Assessed for eligibility (n=64)

**Supplementary Figure 1.** Participant enrollment flow chart

No effect of resveratrol supplementation after 6 months on insulin sensitivity in overweight adults: a randomized trial

M. de Ligt et al.; On-line Supplementary Material

**

**

**Supplementary Figure 2.** Schematic presentation of the study design.

Test day 1 and 2 are identical pre- and post-intervention. OGTT, oral glucose tolerance test

No effect of resveratrol supplementation after 6 months on insulin sensitivity in overweight adults: a randomized trial

M. de Ligt et al.; On-line Supplementary Material

**Supplementary Table 1.** Results of one-way repeated measured ANOVA for the effects of time and treatment on body weight and physical activity score

| **Variable** | **Effect** | **df** | **MS** | **F** | **P-value** |
| --- | --- | --- | --- | --- | --- |
| Body weight | Time | 1.732 | 4.162 | 1.787 | 0.180 |
|  | Time*treatment | 1.732 | 1.186 | 0.509 | 0.577 |
| Physical activity score | Time | 1.968 | 0.330 | 0.478 | 0.619 |
|  | Time*treatment | 1.968 | 0.506 | 0.733 | 0.482 |

Results of a one-way repeated measured ANOVA with Greenhouse-Geisser correcting. Data are presented as the dependent variables, effect, degrees of freedom (df), mean of squares (MS), F- and P-values. The dependent variables were measured at three time-points: pre-intervention, mid-intervention and post-intervention. Treatment conditions were resveratrol (n=20) or placebo (n=21) for six months.
